# Aspartylglycosaminuria: a review

**DOI:** 10.1186/s13023-016-0544-6

**Published:** 2016-12-01

**Authors:** Maria Arvio, Ilkka Mononen

**Affiliations:** 1 Päijät-Häme Social Welfare & Healthcare Joint Municipal Board, Lahti, Finland; 2 KTO, The Special Welfare District of Southwestern Finland, Paimio, Finland; 3 PEDEGO Research Unit, Oulu University Hospital, Finland, Oulu, Finland; 4 Newborn Screening Center Finland, Saske, Turku University Central Hospital, Turku, Finland; 5 The Joint Clinical Chemistry Laboratory at Turku University Hospital, Turku, Finland; 6 Department of Clinical Chemistry, University of Turku, PO Box 52, FIN-20521 Turku, Finland

**Keywords:** Aspartylglucosaminuria, Aspartylglycosaminuria, Lysosome, Lysosomal strorage, Aspartylglucosamine, Glycoasparagine, Glycosylasparaginase, Aspartylglucosaminidase, Glycoprotein degradation

## Abstract

Aspartylglucosaminuria (AGU), a recessively inherited lysosomal storage disease, is the most common disorder of glycoprotein degradation with a high prevalence in the Finnish population. It is a lifelong condition affecting on the patient’s appearance, cognition, adaptive skills, physical growth, personality, body structure, and health. An infantile growth spurt and development of macrocephalia associated to hernias and respiratory infections are the key signs to an early identification of AGU. Progressive intellectual and physical disability is the main symptom leading to death usually before the age of 50 years.

The disease is caused by the deficient activity of the lysosomal enzyme glycosylasparaginase (aspartylglucosaminidase, AGA), which leads to a disorder in the degradation of glycoasparagines – aspartylglucosamine or other glycoconjugates with an aspartylglucosamine moiety at their reducing end – and accumulation of these undegraded glycoasparagines in tissues and body fluids. A single nucleotide change in the AGA gene resulting in a cysteine to serine substitution (C163S) in the AGA enzyme protein causes the deficiency of the glycosylasparaginase activity in the Finnish population. Homozygosity for the single nucleotide change causing the C163S mutation is responsible for 98% of the AGU cases in Finland simplifying the carrier detection and prenatal diagnosis of the disorder in the Finnish population. A mouse strain, which completely lacks the *Aga* activity has been generated through targeted disruption of the *Aga* gene in embryonic stem cells. These *Aga*-deficient mice share most of the clinical, histopathologic and biochemical characteristics of human AGU disease. Treatment of AGU mice with recombinant AGA resulted in rapid correction of the pathophysiologic characteristics of AGU in non-neuronal tissues of the animals. The accumulation of aspartylglucosamine was reduced by up to 40% in the brain tissue of the animals depending on the age of the animals and the therapeutic protocol. Enzyme replacement trials on human AGU patients have not been reported so far. Allogenic stem cell transplantation has not proved effective in curing AGU.

## Background

Aspartylglykosaminuria (AGU) has been found to be the most common recessively inherited disease in the Finnish population. It has been described in early 1960’s and so far approximately 260 Finnish patients and single patients representing all races and several nationalities have been identified with this genetic defect. The clinical, molecular genetic and biochemical research data is reviewed in this paper for this often poorly recognized but still rather commonly occurring disability. Practical implications of the available knowledge onto everyday patient care are also outlined.

## Review

### Disease name/synonyms

- Aspartylglucosaminuria (AGU)
- Aspartylglycosaminuria (AGU)
- Glycosylasparaginase deficiency
- Aspartylglucosaminidase deficiency
- AGA deficiency
- Glycoasparaginase deficiency

## Definition

Aspartyglycosaminuria (AGU) was first described in 1968 in a British family with two seriously intellectually disabled children, who excreted large amounts of aspartylglucosamine (2-acetamido-1-(β’-L-aspartamido)-1,2-didieoxyglucose; GlcNAc-Asn) in their urine [1]. At the same time in Finland, a screening study concerning amino acids in urine among 2177 intellectually disabled individuals identified 11 patients with similar clinical symptoms and excretion of large amounts of an unknown compound containing aspartic acid in their urine [2]. The combined evidence from the Finnish and British patients demonstrated that all of them suffered from AGU. Eventually this led to the realization that enrichment of AGU had occurred in the Finnish population.

AGU is the most common lysosomal storage disease caused by a disorder in degradation of glycoproteins. Glycoproteins commonly contain carbohydrate moieties, which are joined to protein moiety by means of an N-glycosidic bond between amino acid L-asparagine and monosaccharide N-acetylglucosamine. AGU is caused by deficient activity of the lysosomal enzyme glycosylasparaginase that cleaves the N-glycosidic bond between the L-asparagine and N-acetylglucosamine moieties of GlcNAc-Asn. This enzyme deficiency results in the accumulation of undegraded aspartylglucosamine and a series of other glycoasparagines i.e., glycoconjugates containing an L-asparagine moiety linked to the carbohydrate chain in tissues and body fluids of the affected [3, 4].

## Population genetics

AGU belongs to the group of disorders referred to as the Finnish disease heritage [3–6]. Approximately 260 patients have been reported in Finland (2014 population: 5.3 million), and 160 of those are currently alive. Approximately 200–300 living AGU patients are currently known worldwide. Approximately 30 patients are known to be living in Sweden and Norway, and at least some of them are descendants of Finns [7]. AGU frequency of 1 in 3643 was found in a study of children in eastern Finland corresponding to a carrier rate of 1 in 30 in that population [8]. In about 98% of the Finnish AGU patients, the disease is caused by a single point mutation in the glycosylasparaginase gene (see later). It is estimated that the carrier of this mutation is 1 in 50–60 among Finns [9, 10]. Annually, 1–3 children with AGU are born in Finland implying a prevalence of 1.7–5/100.000 live births in its population. Single, but increasing number of AGU patients have been reported globally and in all races suggesting that the disease may be underdiagnosed. In many of these patients the disease-causing gene mutation is different from that of common in the Finnish population (see later).

## Clinical description

AGU is a neuropsychiatric, generalized disease with a progressive course affecting the whole body and resulting in premature aging. A detailed clinical picture according to the age covering the patients’ cognition, speech, self-help, motor skills, personality, growth, mouth, head and face, skin, connective tissue, state of health, as well as laboratory and X-ray findings is given in Tables 1 and 2 [11–13]. The speed of progression of AGU varies between separate patients being a little slower among females.

**Table 1:** Progressive nature of AGU: Growth, cognition, personality, speech, self-help, and motor skills according age. ID = intellectual disability

Age in years	Growth	Cognition	Personality	Speech	Self-help	Motor skills
<2	Early growth spurt +1- +2sd	Normal	“An easy baby”	Normal	Normal	Stiffness in hips
2–5	+1SD	Subnormal	Well-behaved and fussy	Delayed	Delayed	Walks clumsily
6–9	0- + 1sd	Mild ID	Talkative, kind, stubborn	Unclear	Delayed	Can bike and ski, not skate
10–15	Slight and short pubertal growth spurt, early menarche, macro-orchidism	Moderate ID	Fond of children, joyful	Clear	Independent in toileting, dresses and undresses	No change
16–19	Growth ceases, -1SD	Moderate/severe ID	Tends to withdraw	“Soft”	Able to do little shopping	No change
20–24	Girls gain weight	Severe ID	Attached to parents, shows no interest to opposite sex	“Soft”	Able to move outdoors in familiar surroundings	No more biking nor skiing
25–34	No change	Severe ID	Calm, passive	Vocabulary decreases	Active and passive periods	May walk without a goal
35–44	No change	Severe ID	Confused	Few words	Constant need of help	Legs seem not to respond
45+	Loss of weight	Profound ID	Sits still for hours, angry when disturbed	No speech	Constant need of help	Poor balance/wheel chair

**Table 2:** Progressive nature of AGU: mouth, head and face, skin, connective tissue, health and test findings according age

Age in years	Mouth	Head and Face	Skin	Connective tissue	State of health	Test findings, brain MRI
<2	Normal	Macrocephalia, broad mandiple, short and broad nose	Facial erythema	Hernia, planovalgus, clubfoot	Respiratory infections, diarrhoea	Vacuolated cells in all tissues
2–5	Large tongue, broad dental arches, food retention	Generous cheeks, periorbital fullnes	Piezogymic papules in heels, white spots	Tapered fingers, lordosis	Respiratory infections, diarrhoea	Delayed myelination, decreased T2 signal intensity of the thalami
6–9	Gingivitis, oral candida	No change	No change	Bulging abdomen, knock-knees	Benign subcutaneous tumors	Thick and misshapen ribs, vertebral dysplasia
10–15	Gingival overgrowts	No change	Facial seborrhoea	Broad and low ball of foot	Arthritis rheumatoides	Decreased T2 pulvinar signal intensity, mild cerebral atrophy
16–19	Edemic cheeks, cross bite	No change	Angiokeratoma	Thoracic deformity	Psychotic periods, epilepsy	Neutropenia, thrombopenia, mild cerebellar atrophy
20–24	No change	Coarsening facial feature	Facial angiofibromas	Childish appearance	Restless sleep, confusion periods	Evident cerebral and cerebellar atrophy
25–34	No change	Thick eyebrows	Facial rosacea	Poor carriage	Epilepsy	–
35–44	Loss of teeth and loss of gingical overgrowts	Thick and broad/full lips	Loose skin	Muscle atrophy and hypotony	Bursitis, osteoporosis, orofacial	Attenuation in EEG
45+	Drooling	Microcephalia	Angiofibromas and rosacea increse	Contractures in fingers and elbows	Abscesses, fistula of skin, diarrhoea, anaemia, hearth insufficiency, p	Progression of cerebral and cerebellar atrophy

**Fig. 1:**
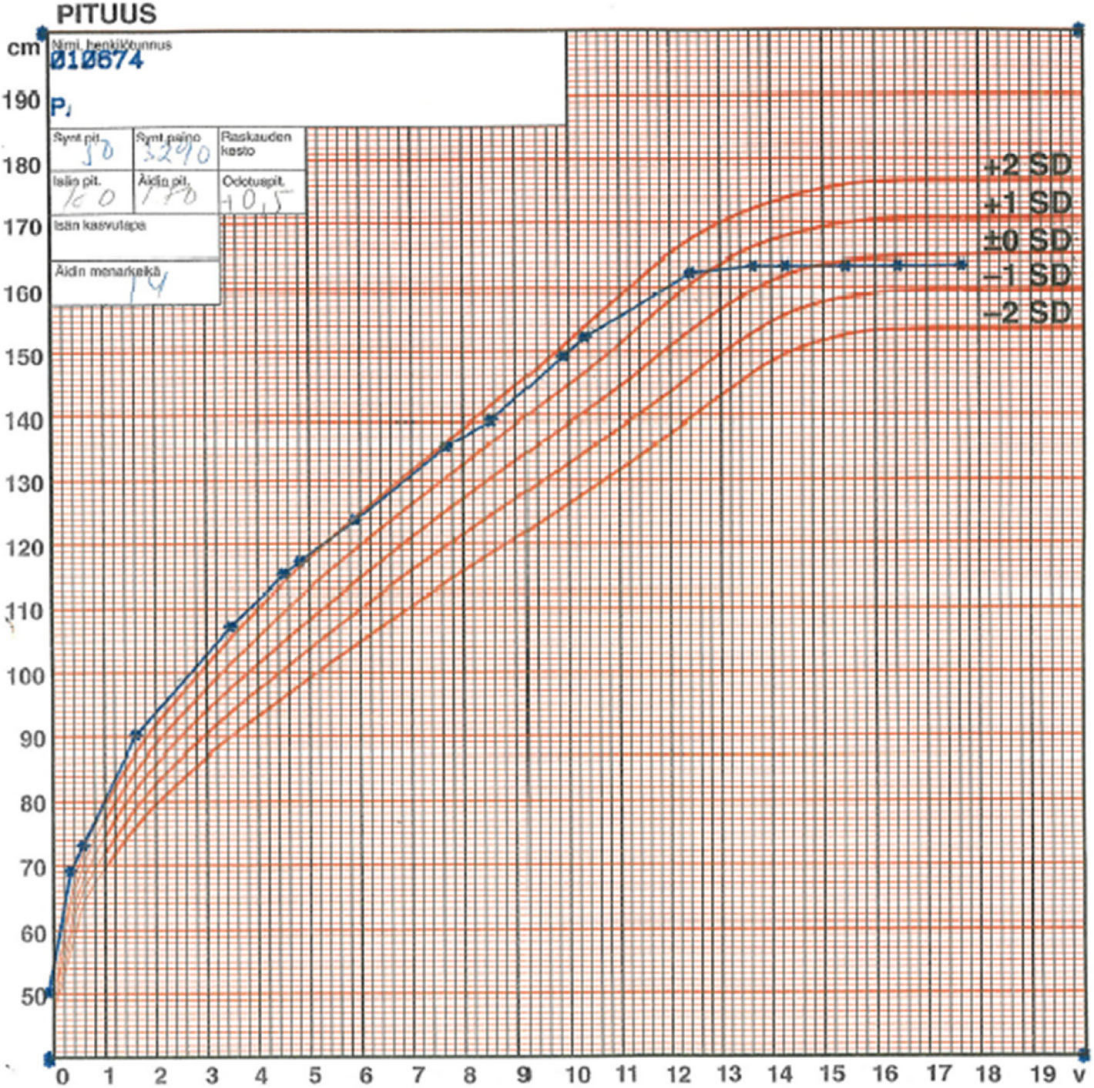
A growth curve of a female AGU patient demonstrates excessive infantile growth, early but weak pubertal growth spurt, and reduced adult height

**Fig. 2:**
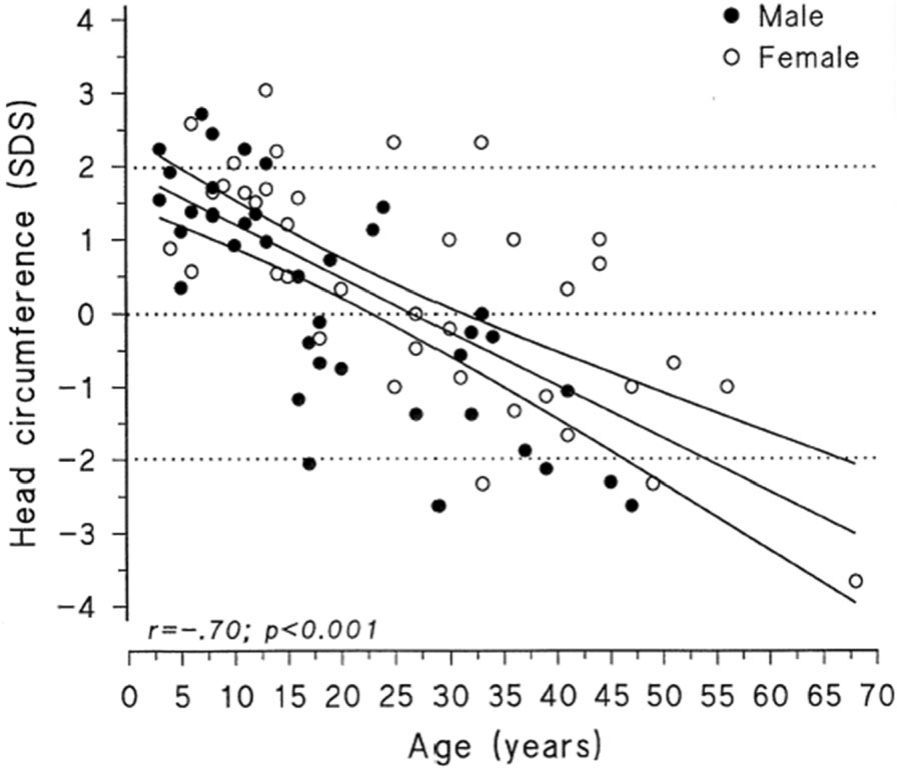
Comparison of head circumferences (SD) of 78 AGU patients and Finnish normative data according chronological age shows macrocephaly in children and microcephaly in adults

**Fig. 3:**
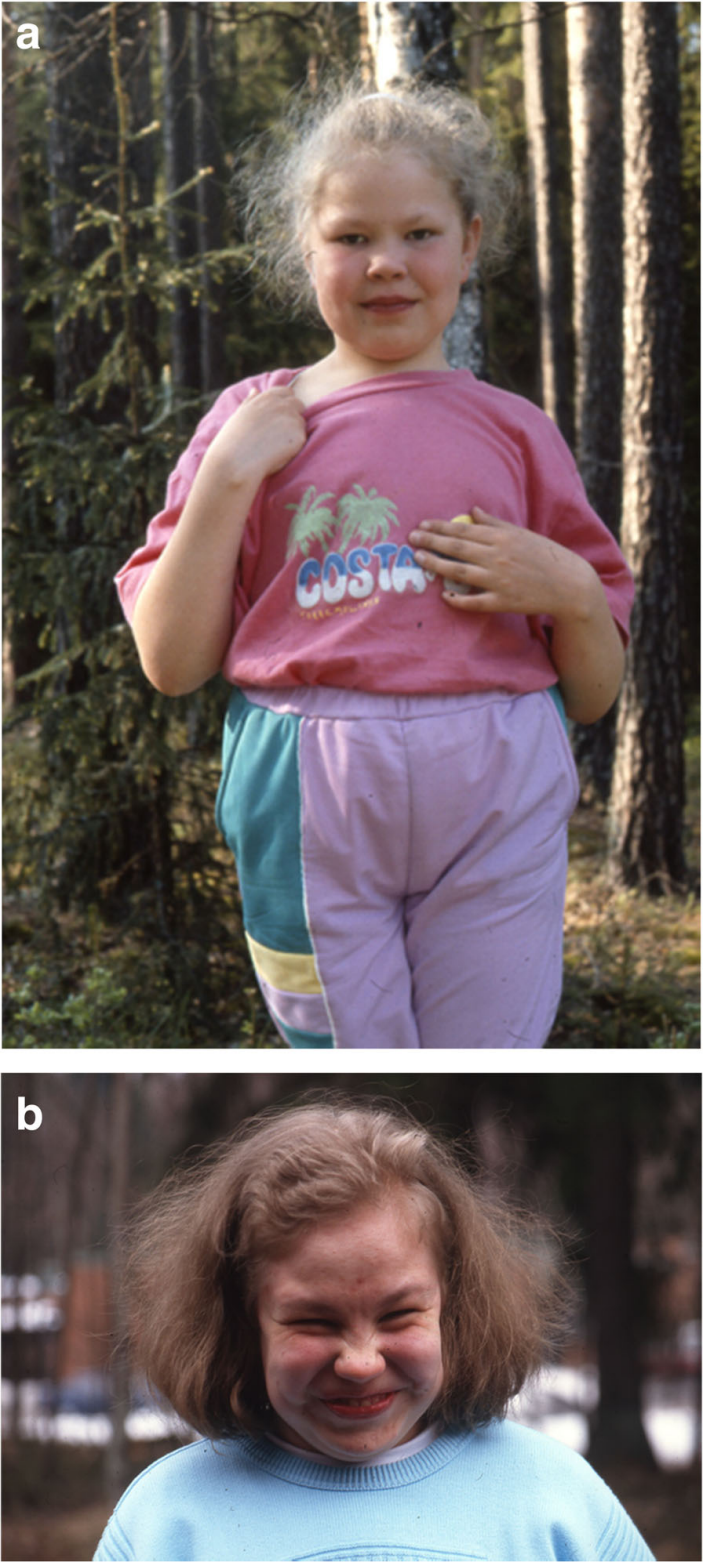
**a**, **b** Consistent dysmorphic gestalt in AGU consists of hypertelorism, puffy eyelids, a short and broad nose with round nares

At birth, the children with AGU are usually healthy with normal birth measurements. An infantile growth spurt (Fig. 1) and increased head circumference (Fig. 2) are the first symptoms [14] associated to hernias and respiratory infections are the key signs to an early identification of AGU. In childhood AGU appears as delayed speech development, poor motor coordination and exceptional placidity or periods of hyperactivity [15]. On the average, the clinical diagnosis of AGU is made at the age of 5 years. The developmental profile comprises three phases: i) Firstly, an abnormally slow but positive development up to the age of 13–16 years, ii) a stable period with a mild and slow decline up to the age of 25–28 years, and finally iii) a rapid decline after the age of 30 years. In psychological tests, the under school-aged AGU children score at a subnormal intellectual disability level, school-aged children at a mild intellectual disability level, teenagers and young adults at a moderate level, adults at a severe level, and the middle aged at a profound intellectual disability level [16, 17]. The patient’s appearance, facial features and body shape as well as their personality are characteristic to the disease (Fig. 3 a-b) [18]. The oral health is impaired in almost all adult patients. Every third patient suffers from epileptic seizures (often nocturnal frontal lobe epilepsy), every fifth from psychiatric disorders and every 20^th^ from rheumatoid arthritis as an associated condition [19–23]. There are many laboratory and imaging findings characteristic to AGU, which are of no clinical significance. It is useful to be aware of these so that the patient is not referred for further examination, for example bone marrow aspiration due to a low white blood cell count in their peripheral blood. Ribs are thick and misshapen, while long bones are thin. The vertebra may show skeletal abnormalities. The skull is often thick and as the patient ages, the loss of brain cells is evident. The disease has a characteristic MRI finding [24]. A special phenomenon is the reduction or shrinking of head circumference as the patient ages (Fig. 2) [25].

## Molecular genetics

The primary defect in AGU is a mutation in the AGA gene comprising 9 exons and located on 4q34.3. The AGA sequence is highly conserved in evolutionary terms [26].

In the Finnish patients with AGU Mononen et al. [27], Fisher and Aronson [28] and Ikonen et al. [29] independently identified homozygosity for two point mutations (G482A and G488C) in the cDNA of glycosylasparaginase. The first nucleotide change results in the replacement of arginine by glutamine (R161Q) and the second change results in a cysteine-to-serine substitution (C163S) in the glycosylasparaginase enzyme protein. Site-directed mutagenesis and expression studies in COS cells demonstrated that only the cysteine to serine substitution (C163S) causes the deficiency of glycosylasparaginase activity [30]. The mutation destroys a disulfide bond [31] and leads to conformational changes in the inactive prercursor enzyme protein preventing its autocleavage into subunits and an active enzyme protein [32].

Screening of 115 Finnish AGU patients demonstrated that 98% of them were homozygous carrying the same two nucleotide changes (G482A and G488C) [33]. Thus, this so called AGU_FIN_ major mutation is a result of a founder effect and demonstrates a high molecular homogeneity in the AGU alleles in the Finnish population. A genealogical study among nine AGU patients from seven families living in northern Norway, who were homozygous for the AGU_FIN_ major mutation, proved Finnish ancestry in all pedigrees. These Finnish immigrants originated from northern Finland in a continuous immigration movement from 1700 to 1900. The second AGU causing allele in the Finnish patients is a 2 base pair deletion in exon 2 of the gene (AGU_FIN_ minor mutation) contributing to approximately 1.5% of the Finnish patients [34].

The data from the Human Gene Mutation Database (HGMD) [35] comprise currently more than 30 different AGU disease-causing mutations in the AGA gene. Half of them are missense mutations. The rest of them include small deletions and insertions, splicing mutations, gross deletions and complex rearrangements in the AGA gene. No genotype/phenotype correlations have been described so far.

## Glycosylasparaginase and pathophysiology of AGU

AGA encodes an L-asparaginase (glycosylasparaginase), which catalyzes the hydrolysis of aspartylglucosamine and other glycoasparagines with s series of larger carbohydrate chains in lysosomes [3]. Glycosylasparaginase removes the L-asparagine moiety from glycoasparagines and L-aspartic acid, ammonia and free N-acetylglucosamine or a carbohydrate chain is generated, respectively. Both the β-amino and β-carboxyl group of the L-asparagine moiety of the substrates must be free in order to enable their anchorage in the active site of glycosylasparaginase and their hydrolysis to occur [36, 37]. Therefore, complete proteolysis of the polypeptide chain surrounding each L-asparagine amino acid carrying an N-glycosidic oligosaccharide moiety must always occur before the hydrolysis of the GlcNAc-Asn linkage. Glycolasparaginase also acts as an L-asparaginase and catalyzes the hydrolysis of free L-asparagine to L-aspartic acid and ammonia [38]. Based on the mechanism of action of glycosylasparaginase, a sensitive fluorometric to measure its activity has been developed using L-aspartic acid β (7-amino-4-methylcoumarin) as the substrate [39]. The combined evidence indicates that glycosylasparaginase reacts specifically toward the L-asparagine moiety of its substrates cleaving it with a mechanism of action similar to bacterial L-asparaginases. Thus the term “glycosylaspaganise” best describes its mode of action.

Glycosylasparaginase belongs to a superfamily of enzymes called as N-terminal nucleophile hydrolases [40, 41], since it catalytically uses a processed N-terminal threonine (Thr 206) [27, 36] as both a polarizing base and nucleophile to act on its subtrates.

The deficient activity of glycosylasparaginase activity results in accumulation of undegraded aspartylglucosamine and other glycoasparagines in body fluids and tissues of AGU patients [3] and AGU mouse model [42]. Hypertrophied storage lysosomes are present in all tissues and organs, but their morphology or histochemical properties are not diagnostic to distinguish the disease from many other lysosomal storage disorders.

## Animal model

Research into the pathogenesis and therapy of AGU in humans was hampered until an animal model for the disease was developed through targeted disruption of the mouse *Aga* gene in embryonic stem cells [42]. The *Aga*-null – *Aga* (-/-) -mice completely lack glycosylasparaginase activity resulting in massive accumulation of aspartylglucosamine in their body fluids and tissues. The level of accumulation of GlcNAc-Asn in the Aga (-/-) mice is very comparable to that found in urine and tissues of human AGU patients.

Histopathological analyses using both light and electron microscopy demonstrated lysosomal hypertrophy in all visceral organs resembling that of human AGU. In the central nervous system (CNS), vacuolized neurons, glial cells and endothelial cells were observed in all regions studied including frontal cortex, cerebellum, brain stem and spinal cord. As a conclusion, the histological features including the distribution and appearance of the hypertrophic lysosomes of the Aga (-/-) closely resemble those in human AGU patients [43].

The early development of Aga (-/-) mice, like that of human AGU patients, did not show any significant phenotypic features. Starting at the age of 6 months, a gradual deterioration in condition leading to impaired neuromotor coordination, bladder function and premature death was observed in *Aga* (-/-) mice [44].

## Diagnosis and diagnostic methods

Vacuolated cells, as in other lysosomal storage diseases, with expanded lysosomes can be detected in all AGU patient tissues in microscopic examinations. Biochemical diagnosis AGU is based on the examination of urinary oligosaccharides [45–47] and assay of glycosylasparaginase activity. Demonstration of accumulated aspartylglucosamine [48, 49] and other glycoasparagines in urine must lead to the measurement of glycosylasparaginase activity e.g., in serum, leukocytes or fibroblasts [50, 51]. The enzyme assay in cultured amniotic fluid cells or chorionic villus samples [52, 53] or directly in amniotic fluid enable prenatal detection of the disease. The analysis of amniotic fluid glycoasparagines may not permit reliable diagnosis of AGU [54]. Analysis of urinary aspartylglucosamine or other glycoasparagines, or glycosylasparaginase activity e.g., in leukocytes [51], however, do not allow reliable carrier detection of AGU, but DNA tests are needed for that purpose. There are DNA tests available to detect the AGU_FIN_ major mutation [27–29] and AGU_FIN_ minor mutation [34] and carriers of those mutations [55, 56].

## Differential diagnosis

An early growth spurt (Fig. 1) and development of macrocephalia (Fig. 2) followed by delayed speech development, physical clumsiness and exceptional placidity or periods of hyperactivity in a child with AGU-characteristic facial features (Fig. 3) usually rises a suspicion of AGU in Finland where the disease is well-known among child neurologists. The early signs in other diseases manifesting as intellectual disability included in the Finnish disease heritage, such as Salla disease, neuronal lipofuscinoses and Northern epilepsy, appear in another way. Poor eye contact, nystagmus, and ataxia associated to general muscle hypotony are the first signs in Salla disease; impaired vision in different types of neuronal lipofuscinoses; and epileptic seizures in Northern epilepsy.

The presence of vacuolated lymphocytes in peripheral blood and dilated, large lysosomes in tissue cells of a patient may be the first finding to indicate a lysosomal storage disease. A careful examination of the histology specimens may indicate whether the accumulating compound might be composed of lipids, glycosaminoglycas or oligosaccharides suggesting the further studies to identify the storage material. All disorders of glycoprotein degradation – including AGU – result in accumulation of oligosaccahrides in body fluids and tissues. Analysis of oligosaccharides in urine is best way to screen these disorders (see earlier). The same applies to such lysosomal storage disorders as certain types of gangliosidoses, sialic acid storage disorders and I-cell disease. Glycosaminoglycans in urine should also be analyzed to rule out various types of mucopolysaccharodoses. The identification of the structure of the accumulated material leads to specific enzyme analyses and the lack of the activity of a particular lysosomal enzyme activity finally confirms the correct diagnosis of the lysosomal disease. The assay of the activity of the missing enzyme – glycosylasparaginase - can be used to diagnose other potential AGU patients in the family or prenatal testing. The sequence analysis of the cDNA or gene of the affected enzyme protein often reveals the mutation behind the structural defect in the enzyme protein and the analysis of this mutation can be used e.g., to carrier detection, prenatal diagnosis or diagnosis other affected individuals in the family.

## Management and treatment

It is generally accepted that the intracellular accumulation of storage material –glycoasparagines – in the deficiency of glycosylasparaginase leads to the multisystem AGU disease characterized by progressive psychomotor retardation and structural changes in various tissues. Thus it is reasonable to assume that timely correction of the intracellular activity of glycosylasparaginase would prevent of the accumulation of glycoasparagines and thus the progression of the clinical symptoms of the AGU patients would cease and potentially reverse.

There is currently no curative treatment for human AGU disease. Some patients in Finland and Sweden have undergone bone marrow transplantation with no benefit [57, 58]. Associated impairments like epileptic seizures, psychotic periods and arthritis are treated according general therapeutic guidelines. Carbamazepine has been used as a drug of choice effective in the treatment of epilepsy. The aim of rehabilitation and education is as proficient as possible mastery of social and independent living skills and good self-esteem. The rehabilitation plan is build individually and most often includes training at kindergarten and a special school for children with developmental disorders, speech and occupational therapy during childhood. Adult AGU patients often benefit from physio- and music therapy.

## Experimental therapy

In vitro experiments demonstrate that the metabolic defect in AGU fibroblasts and lymphocytes can effectively be corrected by enzyme replacement therapy with recombinant human glycosylasparaginase [59]. Correction of intracellular glycosylasparaginase activity to the level of 3–4% of that in normal lymphocytes cleared cultured human AGU lymphocytes from intracellular aspartylglucosamine stores and its further accumulation was prevented. Cell-to-cell transfer of glycosylasparaginase from normal to AGU cells occurs [60].

Recombinant human glycosylasparaginase is biologically active also in the experimental enzyme replacement therapy of the AGU mouse model [61]. Pathophysiologic characteristics of AGU were effectively corrected in non-neuronal tissues of adult AGU mice during 2 weeks of AGA therapy. At the same time, glycosylasparaginase activity increased to 10% of that in normal brain tissue and aspartylglucosamine amount was reduced by 20% in total brain of the treated *Aga* (-/-) mice. AGA therapy in newborn mice was even more effective reducing the amount of stored aspartylglucosamine in the brain tissue up to 40%. The response to therapy was dose-dependent: the higher the glycosylasparaginase dose the faster the clearance of aspartylglucosamine [62]. Massive accumulation of a larger glycoasparagine (Man2GlcNAc-Asn) in tissues of AGU mice was also effectively corrected with glycosylasparaginase replacement therapy [63].

Interestingly, added glycosylasparaginase can induce apoptosis of L-asparagine-dependent leukemia cells in vivo by depleting the cells from their L-asparagine stores [64].

## Fetus and newborn

AGU has been diagnosed prenatally demonstrating the deficiency of glycosylasparginase activity in cultured amniotic fluid cells [52, 53]. Electron microscopic evidence of lysosomal storage was observed in several organs including kidneys of the fetus affected by AGU. Elevated amount of aspartylglucosamine in the midterm amniotic fluid from the pregnancy with the fetus affected by AGU further demonstrates of the presence of the metabolic defect of AGU prior to birth. Neonatal detection of AGU can be accomplished demonstrating lacking glycosylasparaginase activity in umbilical cord serum samples [65].

## Prognosis

Table 1 summarizes various effects of the missing activity of glycosylasparaginase on the AGU patients. An AGU disease carrier may have a higher risk of arthritis and their facial bones show features characteristic to AGU disease [5, 18]. Life expectancy for Finnish female patients is on the average 50 years and for men 45 years. The oldest known patient lived to the age of 69 years. Before death the patients usually experience a period of inactivity, poor general condition and fatigue. A dystonic status refractory to treatment may last for several days just before death. Autopsy findings if Finnish AGU patients suggest that the patients usually die of severe bacterial infections, mostly pulmonary infections.

## Future aspects

Treatment of various lysosomal diseases has made a marked progress during the past two decades. The development of a variety of innovative therapeutic approaches include increasing the residual activity of the missing enzyme by stem cell transplantation, pharmacological chaperons, combination of chaperons and enzyme replacement therapy, substrate reduction therapy and gene therapy (for references [66]). Experience from these therapies will be beneficial in tackling the main limitations associated to enzyme replacement therapy in lysosomal diseases such as high cost, immunologic response and lack of efficacy to central nervous system. They represent significant challenges for future development of new drugs for treatment of AGU as well.

## Conclusions

AGU studies have been conducted in many countries and new patients representing non-Finnish AGU mutations are frequently found. Although there is no curative treatment for this progressive disease it is considered important to share the information of its clinical course. The patients and their families can be supported in many ways, i.e., by providing both medical, educational and social services during the patient’s life span. Most health issues associated with AGU, e.g., like epilepsy, rheumatoid arthritis, and infections, can be treated according to general guidelines. On the other hand, it may help families to overcome the difficult phases of disease’s course to know that the stressful, drug-refractory manic-apathetic periods, which are known to affect about 20% of patients during their adolescent years, usually subside in young adulthood. Also, physiotherapy and other therapies thorough life can often be beneficial. The great importance of peer support of the family association should not be forgotten either. From the socio-educational perspective the burden of parents can be eased by individually tailored learning goals in early childhood and in adolescence by organizing suitable daily activities in group homes and/or day care centers. Later on most of the diseased persons need a nursing home. Once the special needs of the patients are met and treated, they themselves are usually quite satisfied with their daily living. Successful therapy of AGU will require therapeutic strategies, which effectively increase glycosylasparaginase activity in central nervous system.

## Abbreviations

AGA: Glycosylasparaginase, Aspartylglucosaminidase
AGU: Aspartylglucosaminuria, Aspartylglycosaminuria
GlcNAc-Asn: 2-acetamido-1-(β’-L-aspartamido)-1,2-didieoxyglucose, Aspartylglucosamine
MRI: Magnetic resonance image
